# Differences between individuals who left a suicide note and those who did not: a systematic review

**DOI:** 10.1192/j.eurpsy.2026.10688

**Published:** 2026-07-31

**Authors:** G. Gotti, A. Bordessa, F. Turolla, S. Tambuzzi, G. Gentile, R. Zoja, F. Madeddu, L. Montali, R. Calati

**Affiliations:** 1 Department of Psychology, University of Milan-Bicocca , Milan, Milan; 2 Department of Translational Medicine, University of Eastern Piedmont, Novara; 3 Department of Biomedical Sciences for Health, Section of Legal Medicine, University of Milan, Milan, Italy; 4 Department of Adult Psychiatry, Nîmes University Hospital, Nîmes, France

## Abstract

**Introduction:**

Suicide notes provide unique insight on the cognitive and emotional state of suicide victims in their final moments. However, not everyone who dies by suicide leaves a note. Thus, it remains uncertain whether note-writers (NW) constitute a representative subgroup of those who died by suicide.

**Objectives:**

This systematic review aimed to provide a synthesis of literature examining the different characteristics between NW and non-note-writers (NNW).

**Methods:**

A literature search was independently performed by three authors using the PubMed and PsycINFO databases (up to June 6^th^, 2025). Studies were included if they compared NW and NNW in terms of sociodemographic (e.g. gender, age, marital status), clinical (e.g. mental or physical illness, use of alcohol or drugs), or suicide-related variables (e.g. method of suicide, previous suicidal behavior) between NW and NNW. These results are preliminary, as article screening is still ongoing.

**Results:**

The search initially identified 852 references. Twenty-six studies met the inclusion criteria and were included. Regarding sociodemographic variables, 38% (k=10) of studies found gender differences: NW were more frequently males in half of these, females in the other half. Age differences emerged in 27% (k=7) of total studies: in most cases, younger people more frequently left notes, while middle-aged and older people did in fewer studies. Marital status differed between NW and NNW in 27% (k=7) of total cases: across studies NW were more frequently separated or divorced, single, or widowed. In 19% (k=5) of total studies, NW had greater financial problems than NNW. Concerning clinical variables, mental illnesses were less frequent in NW in 23% (k=6) of studies. Regarding suicide-related variables, NW differed from NNW for the methods for suicide in 65% (k=17) of studies: poisoning, gas intoxication, and drugs were more common in NW than in NNW. History of suicide attempts or suicidal behavior differed in 19% (k=5) of studies: it was more frequent in NW in most cases, less frequent in a few of them. Other variables were significantly different in 4 or less studies.

**Conclusions:**

Available evidence on differences between NW and NNW is heterogeneous and inconclusive. Some variables appear to distinguish the two groups, but diversity of results and study characteristics—such as sample size, participant profiles, and research settings—cautions against generalizing findings from NW to the broader suicidal population.

**Disclosure of Interest:**

None Declared

